# Medication Regimen Complexity and Medication Burden Among Patients With Type 2 Diabetes Mellitus: A Retrospective Analysis

**DOI:** 10.3389/fphar.2022.808190

**Published:** 2022-03-21

**Authors:** Norazida Ab Rahman, Ming Tsuey Lim, Shantini Thevendran, Najwa Ahmad Hamdi, Sheamini Sivasampu

**Affiliations:** ^1^ Institute for Clinical Research, National Institutes of Health, Ministry of Health, Selangor, Malaysia; ^2^ Public Health Development Division, Ministry of Health, Putrajaya, Malaysia

**Keywords:** medication regimen complexity, MRCI, medication burden, glycemic control, type 2 diabetes mellitus, polypharmacy, medication adherence, primary care

## Abstract

**Introduction:** Most type 2 diabetes mellitus (T2DM) patients with chronic conditions require multiple medications to achieve and maintain good glycemic control.

**Objective:** This study assessed medication burden, regimen complexity, and adherence among T2DM patients and evaluate its association with glycemic control.

**Method:** We analyzed data of 2,696 T2DM patients at public health clinics in Malaysia from January 2018 until May 2019. Medication burden was based on medication count, regimen complexity was measured using the validated Medication Regimen Complexity Index (MRCI) tool, and adherence was measured using proportion of days covered (PDC) formula. Logistic regression models were used to compute unadjusted and adjusted odds ratio (aOR) with 95% confidence interval (CI) for association between the medication parameters and glycemic control (HbA1c ≤ 7.0%) over a 90-day period.

**Results:** The cohort mean age was 60.4 years old (±10.8) and 62.9% were female. Overall, the average medication count was 4.8 with MRCI score of 15.1. Mean adherence score (PDC) was 90%. High medication count and MRCI scores were associated with lower odds of achieving good glycemic control (aOR 0.88; 95% CI 0.82, 0.94 and aOR 0.89; 95% CI 0.87, 0.92, respectively) while inverse association was observed between adherence and HbA1c level (aOR 2.7, 95% CI 1.66, 5.19). Similar findings were observed for diabetes-specific measures.

**Conclusions:** High medication count, high regimen complexity, and low medication adherence were associated with poor glycemic control over the 3-month follow-up period. These parameters could be used to identify patients with complex pharmacotherapy regimens so that targets for intervention can be taken to achieve optimum outcomes and ease of self-care.

## Introduction

Diabetes is a chronic illness that requires long-term medical care and patients are usually prescribed medications when attempts at lifestyle modifications alone fail to achieve or maintain adequate glycemic control. Patients with type 2 diabetes mellitus (T2DM) often requires more than one medication, which grows in complexity as the disease progresses and complications arise (Peron et al., 2015). The prevalence of multiple comorbidities among patients with established T2DM also increases the need for more medications and further complicates the treatment regimen. Besides medication count, other contributing attributes to the complexity of a medication regimen including dosage forms, dosing frequencies, complicated schedules, and special administration directions present challenges for patients with T2DM (Advinha et al., 2014). Studies have reported that complex treatment regimens are associated with worse outcomes, including medication non-adherence, poor control of medical conditions, and increased health resources utilization (Claxton et al., 2001; Ingersoll and Cohen, 2008; Pollack et al., 2010; Pantuzza et al., 2017; Ayele et al., 2019).

In Malaysia, the number of patients with T2DM is steadily increasing; the National Health and Morbidity Survey 2019 estimated that four million adult population had diabetes (Institute for Public Health, 2020). Previous data suggest that on average, patients with chronic condition take five medications per day (Hasan et al., 2017). Among patients with T2DM who attended primary care clinics in Malaysia, 60% reported taking more than three medications (Ahmad et al., 2013). Of note, it was reported that less than 25% of the patients with T2DM achieved the targeted HbA1c levels despite the evidence of improving processes of diabetes care in Malaysia (Mafauzy et al., 2016). One of the possible contributing factors is the medication burden imposed on the patients which resulted in poor adherence and subsequently suboptimal glycemic control (Sav et al., 2015). This has become the main focus in the development of diabetes management strategies, which included simplification of dosing regimens to promote better medication adherence among patients with T2DM (Nieuwlaat et al., 2014). A better understanding of treatment profiles is therefore urgently needed, before proper interventions can be tailored to improve the medication-taking behavior of T2DM patients.

Medication regimen complexity can be quantified using a validated tool, the Medication Regimen Complexity Index (MRCI) (George et al., 2004). The complexity level is based on the medication attributes of the regimen such as the number of medications, prescribed dosage forms, dosage frequency and administration instructions. The MRCI tool has demonstrated good evidence in classifying medication regimen complexity over a simple medication count (Mansur et al., 2012), by discriminating between regimens with the same number of medications but of different complexity. This tool was subsequently expanded and validated to include not only disease-specific prescription medications but also other prescription and over-the-counter (OTC) medications (Libby et al., 2013). The expanded tool, which is referred to as patient-level MRCI, provides a better perspective when patients are at greatest risk for failing to achieve desired outcomes.

To the best of our knowledge, detailed assessment of medication regimen complexity on T2DM has not been evaluated in the primary care settings in Malaysia. Understanding the association between these parameters and glycemic control may help to inform future strategies for developing interventions to improve therapeutic outcomes. The objective of this study was to determine medication burden, regimen complexity, and medication adherence among patients with T2DM and evaluate its effect on the level of glycemic control.

## Methods

Ethical approval was granted by the Medical Research and Ethics Committee, Ministry of Health Malaysia (NMRR-17-267-34768) in compliance with the Declaration of Helsinki. Waiver of consent was also obtained. Medical records were reviewed retrospectively and all data was anonymized before use in the analysis.

### Study Design and Setting

This cohort study was a retrospective analysis of data from a larger study—“The EnPHC interventions and evaluation study (EnPHC-Eva: Facility)”. Pharmacy database from public primary care clinics in Malaysia was also utilized.

Primary care services in Malaysia are provided by a dual healthcare system: government-funded public health clinics and private clinics that operate on a fee-for-service model. Care for chronic diseases is largely concentrated in public health clinics while encounters in private clinics are mostly for acute and minor conditions (Khoo et al., 2015; Sivasampu et al., 2016). Workforce in public clinics encompasses a diverse skill mix including doctors, nurses, pharmacists, assistant medical officers, and other allied health professionals whereas the majority of private clinics are single medical practitioner practices. In public clinics, each clinic has an outpatient pharmacy that supplies and maintain records of medication supply for all patients seen at the respective clinics. Generally, the prescription is valid for the duration until the next scheduled appointment with the doctor. Patients visit the clinics’ pharmacy directly for any prescription refills and the maximum period of each supply is usually limited to 1 month. For late refills, medications can only be dispensed if the prescription is still valid. Patients seeking care at public clinics may also request doctors to prescribe for them to get medications at the private pharmacies, but the proportion is low due to the additional cost incurred.

### Data Sources

Patient and outcome data were obtained from the EnPHC cohort that includes patient demographic and diagnosis at baseline, as well as laboratory parameters collected during subsequent follow-up(s). Details on medication were retrieved from the pharmacy dispensing records. The two databases were linked using a unique patient identifier. Details of the databases are described below.

### The EnPHC Interventions and Evaluation Study (EnPHC-Eva: Facility)

The project “Evaluation of ‘Enhanced Primary Health Care (EnPHC)’ interventions in public health clinics was a quasi-experimental controlled study to assess the effectiveness of an intervention package on the process of care and intermediate clinical outcomes at 40 selected public primary care clinics in Malaysia. The clinics were selected by the study team based on their setup and size that reflects care services provided across public health clinics, along with budget and capacity to implement the EnPHC package. Details about the EnPHC project have been described and published elsewhere (Sivasampu et al., 2020). In summary, patients aged 30 years and older with a recorded diagnosis of T2DM or hypertension were sampled from the participating clinics. Their medical records were reviewed retrospectively and the following data were collected at several time points between November 2016 to June 2019: patient demographic (age, gender, ethnicity), risk factors (comorbidities, disease duration), physical examination (e.g., blood pressure (BP), body mass index), and laboratory investigations (e.g., HbA1c, lipid profile).

### Pharmacy Dispensing Record

The Pharmacy Information System (PhIS) is an electronic medication management system implemented in public primary care clinics throughout Malaysia. The database contains details of medications supplied to patients including prescription date and duration, dispensed date and quantity, medication name, dosage, route, and frequency. Prescription and dispensing data are available from 1 January 2018 onwards.

### Study Population

The present study selected patients with matched records from the two data sources for the period from 1 January 2018 until 31 May 2019. We included patients with T2DM, treated with at least one antidiabetic medication, and had at least one HbA1c reading recorded during this period. All medications prescribed and supplied to patients during the study period were assessed and medications for the treatment of chronic conditions were identified for inclusion in the analysis. Chronic medications were defined as medications with prescriptions filled for a supply duration of 90 days or more (U.S. National Center for Health Statistics, 2018). Patients were excluded if there was no data on medication(s) prescribed for the period before the last HbA1c measurement. The final cohort consisted of 2,696 patients.

### Outcome Measures

Medications were categorized according to the Anatomical Therapeutic Chemical (ATC) classification system recommended by the World Health Organization (WHO) (WHO Collaborating Centre for Drug Statistics Methodology, 2021). Medication regimens for all patients included in the analysis were assessed over 90 days preceding the latest HbA1c measurement. Analysis of medications was carried out at two levels: 1) diabetes-specific which included only antidiabetic medications, and 2) patient-level which included all chronic medications.

### Medication Burden

Medication burden was quantified by simple medication count and calculated as the mean number of chronic medications dispensed per patient, per day. Based on the medication count, patients were grouped into the following categories: 1) one to two medications, 2) three to four medications, 3) five to six medications and 4) ≥ 7 medications.

### Medication Regimen Complexity

Medication regimen complexity was computed using the MRCI tool (George et al., 2004). The validated tool consisted of three sections: dosage forms, dosing frequencies, and additional directions. MRCI score was calculated based on pre-assigned weights for these elements in which a higher MRCI score indicates a more complex medication regimen. Directions for use that were not recorded in the system (e.g. take with food) were identified from medication package insert or drug reference tool (Facts and Comparisons 2016).

### Medication Adherence

Adherence to medications was measured by calculating the proportion of days covered (PDC) over a fixed time interval. PDC is defined as the number of days covered with medications divided by days of observation (Raebel et al., 2013; Pednekar et al., 2019) i.e.,

$$PDC\;=\;\frac{Number\;of\;days\prime \;\mathrm{cov}ered}{Observation\;period}$$

PDC was dichotomized using the convention threshold of 0.8 (Andrade et al., 2006). Patients were considered adherent if they have PDC ≥0.8 (80%).

### Glycemic Control

The main outcome measure was the HbA1c level. Patients’ latest readings of HbA1c were used to categorize them into two groups: controlled = HbA1c ≤ 7.0% and uncontrolled = HbA1c >7.0%. The level was selected based on guideline recommendations of HbA1c target ranges for the general population in Malaysia (Ministry of Health Malaysia, 2015).

### Statistical Analysis

Patient demographics, clinical characteristics, and treatment characteristics were reported descriptively. Association between independent variables (medication burden, medication regimen complexity, and adherence) and dependent variable (glycemic control) were analyzed using binary logistic regression with a dichotomous outcome (HbA1c level ≤7.0% or >7.0%). In multivariable regression analysis, covariates included for adjustment were age, gender, ethnicity, presence of key comorbidities (hypertension, hyperlipidemia), body mass index (BMI), duration since diabetes diagnosis, and location of clinic (urban vs. rural). Covariates included for adjustment in the multivariable model were identified based on univariable analysis and clinical importance. Analysis was carried out using a complex survey design to account for the clustering effect within clinics. Complete case analysis was performed for the regression. Multicollinearity of the covariates was checked.

Sensitivity analysis was conducted to assess the robustness of the results. In the first sensitivity analysis, the HbA1c level was analyzed as a continuous outcome variable and the association was measured using multivariate linear regression. The second sensitivity analysis used a higher HbA1c level of ≤7.5% to dichotomize the outcome of good glycemic control versus poor (HbA1c >7.5%) for measurement of association using logistic regression. The odds ratio (unadjusted and adjusted) and the corresponding 95% confidence intervals (CI) were reported. A *p*-value of <0.05 was considered statistically significant. All statistical analysis was performed using Stata statistical software version 15 (StataCorp, 2017).

## Results

A total of 2,696 patients who met the inclusion criteria were included in the final analysis. Table 1 shows the characteristics of the study population. The mean age of the patients was 60.4 years (range: 30–93 years), 62.9% were females, and 75.1% were of Malay ethnicity. Approximately half of the patients were obese with BMI scores above 27.5 kg/m^2^. Comorbidities of hypertension and hyperlipidemia were present in 85.8% and 74.6% of the patients, respectively. Almost one-third of the patients were treated with a combination of oral antidiabetics with insulin. Only 34.5% had HbA1c below 7.0%.

**TABLE 1:** Demographic characteristic of study cohort from “EnPHC-Eva: Facility” Malaysia, 2018-2019 (N = 2,696).

Characteristics	n (%)
Age (years), mean (SD)	60.4 (10.8)
<65 years	1,744 (64.7)
≥65 years	952 (35.3)
Gender	
Male	1,000 (37.1)
Female	1,696 (62.9)
Ethnicity	
Malay	2,025 (75.1)
Chinese	368 (13.7)
Indian	278 (10.3)
Others	25 (0.9)
Location of primary care clinic	
Rural	1,330 (49.3)
Urban	1,366 (50.7)
BMI (kg/m^2^), mean (SD) (N = 2,610)	28.4 (5.5)
BMI ≥27.5 kg/m^2^	1,367 (52.4)
Comorbidity	
Hypertension	2,312 (85.8)
Hyperlipidaemia	2,011 (74.6)
Duration of diabetes (years), mean (SD)	7.1 (5.7)
Duration of hypertension (years), mean (SD) (N = 2,312)	7.7 (6.5)
Duration of hyperlipidaemia (years), mean (SD) (N = 2011)	5.0 (4.3)
Antidiabetic medication	
Oral anti-diabetic drugs	1,820 (67.5)
Oral anti-diabetic drugs + Insulin	876 (32.5)
Antihypertensive medication	
Free combination antihypertensive drugs	2,297 (99.4)
Fixed dose combination	15 (0.6)
Lipid lowering medication	
Statin	2,348 (87.1)
HbA1c (%), mean (SD)	8.3 (2.1)
Median (IQR)	7.8 (6.7, 9.6)
HbA1c ≤ 7.0%	931 (34.5)
HbA1c > 7.0%	1,765 (65.5)

*Denominator not equal to 2,696 due to missing data.

Abbreviations: BMI, body mass index; HbA1c, glycated haemoglobin; PDC, proportion of days covered; SD, standard deviation.

Table 2 describes treatment characteristics of the patients for three components: medication burden, regimen complexity, and adherence. On average, patients were taking 4.8 chronic medications per day. There were 17% of patients with seven or more medications daily. Antidiabetic medication accounted for approximately 27.1% of the mean total medication burden, with the average antidiabetic medication burden of 1.3. The complexity of the medication regimen was presented as MRCI scores in Table 2 for all chronic medications (patient-level) and antidiabetic medications (diabetes-specific). Breakdown of the individual components of the MRCI was also provided. The overall mean scores of patient-level MRCI and diabetes-specific MRCI were 15.1 and 7.9, respectively. Antidiabetic medications accounted for 52.3% of the overall patient-level MRCI scores. The main contributing attributes to the final score of medication regimen complexity were dosing frequency and the additional instructions given for taking the medications, followed by the dosage forms. In terms of medication adherence, the average PDC was 0.90 at the patient-level and 0.91 for diabetes-specific. Nonetheless, there were 19% of patients with PDC levels below 80% indicating poor adherence.

**TABLE 2:** Treatment characteristics of study cohort from “EnPHC-Eva: Facility” Malaysia, 2018-2019 (N = 2,696).

Variable	n (%)
**Medication burden**	
Total number of medications per day, mean (SD)	4.8 (1.7)
Categories, by quantity	
1–2	221 (8.2)
3–4	942 (34.9)
5–6	1,081 (40.1)
≥7	452 (16.8)
Number of antidiabetic medications per day, mean (SD)	1.3 (0.7)
Percentage of total medication count, %	27.1
Categories, by quantity	
1	1,608 (59.6)
2	935 (34.7)
3–4	153 (5.7)
**Medication regimen complexity**	
Total patient-level MRCI score, mean (SD)	15.1 (4.9)
(i) Dosage form score, mean (SD)	2.0 (1.5)
Percentage of total pMRCI score, %	13.2
(ii) Dosing frequency score, mean (SD)	6.7 (2.4)
Percentage of total pMRCI score, %	44.4
(iii) Additional directions score, mean (SD)	6.4 (2.4)
Percentage of total pMRCI score, %	42.4
Diabetes-specific MRCI, mean (SD)	7.9 (3.0)
Percentage of total pMRCI score, (%)	52.9
(i) Dosage form score, mean (SD)	1.8 (1.2)
Percentage of total dMRCI score, %	22.8
(ii) Dosing frequency score, mean (SD)	3.2 (1.2)
Percentage of total dMRCI score, %	40.5
(iii) Additional directions score, mean (SD)	2.9 (1.3)
Percentage of total dMRCI score, %	36.7
**Medication adherence**	
Patient-level PDC, mean (SD)	0.90 (0.15)
Categories, by PDC level	
<80%	512 (19.0)
80–89%	403 (14.9)
≥90%	1,781 (66.1)
Diabetes-specific PDC, mean (SD)	0.91 (0.15)
Categories, by PDC level	
<80%	471 (17.5)
80–89%	311 (11.5)
≥90%	1,914 (71.0)

Abbreviations: dMRCI, diabetes-specific medication regimen complexity index; PDC, proportion days covered; pMRCI, patient-level medication regimen complexity index; SD, standard deviation.

Table 3 depicts the association between the treatment profiles and good glycemic control (attainment of HbA1c ≤7.0%). Each model was run separately and adjusted for the covariates. For measurement at patient-level (chronic medications), increasing medication count (adjusted OR 0.88; 95% CI 0.82–0.94) and regimen complexity (adjusted OR 0.89: 95% CI 0.87–0.92) was associated with lower odds of achieving HbA1c below 7.0%. On the other hand, increasing adherence was associated with greater odds of achieving target HbA1c (adjusted OR 2.66; 95% CI 1.66–5.19). Similar findings were observed when the analysis was conducted for the diabetes-specific measures. However, the association between adherence and HbA1c level did not reach statistical significance for the diabetes-specific (adjusted OR 1.76; 95% CI 0.86–3.60). Estimates from sensitivity analyses were consistent with the main results (Supplementary Table S1, S2).

**TABLE 3:** Association between medication parameters and HbA1c level (≤7.0%) of study cohort from “EnPHC-Eva: Facility” Malaysia, 2018-2019.

	Unadjusted	Adjusted		
	OR (95% CI)	OR (95% CI)	*p*-value	*R* ^2^
Patient-level				
Medication count	0.92 (0.87, 0.96)	0.88 (0.82, 0.94)	<0.001	0.0862
MRCI score	0.91 (0.89, 0.92)	0.89 (0.87, 0.92)	<0.001	0.1126
Adherence (PDC)	1.91 (1.10, 3.31)	2.66 (1.66, 5.19)	0.004	0.0840
Diabetes-specific				
Medication count	0.54 (0.48, 0.61)	0.59 (0.52, 0.66)	<0.001	0.1003
MRCI score	0.68 (0.66, 0.71)	0.70 (0.67, 0.74)	<0.001	0.1900
Adherence (PDC)	1.11 (0.65, 1.89)	1.76 (0.86, 3.60)	0.123	0.0819

Note: Model was run separately for (i) medication count, (ii) MRCI, score, and (iii) adherence. Estimation using univariate (unadjusted) and multiple logistic regression adjusted for age (years), gender (male/female), ethnicity (Malay/Chinese/Indian/others), hypertension (no/yes), hyperlipidaemia (no/yes), body mass index (kg/m2), location of primary care clinic (rural/urban), duration of diabetes (years). *p*-value significant at <0.05. The variance inflation factors (VIFs) for all variables were less than two in all models. Abbreviations: OR, odds ratio; CI, confidence interval; MRCI, medication regimen complexity index; PDC, proportion days covered.

## Discussion

In this study, we used a validated MRCI tool to quantify the complexity of a medication regimen among patients with T2DM. We found that almost two-thirds of the patients had an average of at least five medications prescribed per day. The diabetes-specific MRCI accounted for 52.9% of the patient-level MRCI score. In our cohort of T2DM patients at public primary care clinics in Malaysia, high medication count, high regimen complexity, and low medication adherence were associated with poor glycemic control over the 3-month follow-up period.

On average, approximately two-thirds of the patients in this study were taking a minimum of five medications per day. This finding is not surprising as approximately 90% of patients with T2DM in our study had at least one other condition such as hypertension, hyperlipidemia or cardiovascular disorders which contributed to the additional medications. Despite the large proportion of patients with a high medication burden, a good medication adherence rate was observed in this study. Several reasons could account for this observation. First, it is possible that the adherence observed in this current study is still good until a certain threshold of medication burden is achieved before it starts to decline. Prior studies suggested that there is a limit to the number of medications a patient can consistently adhere to as prescribed (Shalansky and Levy, 2002; Kim et al., 2019). Second, the adherence measure (PDC) which we have calculated based on medication dispensing data is merely reporting on the rate of medication possession. This limitation may overestimate adherence to treatment for some patients. Finally, good adherence may be partly attributed to patients’ health beliefs in effective disease management (Rosenstock, 2005). In this Health Belief Model, patients who believe that they are ill are likely to make additional efforts to take the medications as prescribed to maintain their health. Of note, medication adherence is a complex behavior. It can be influenced not only by patients’ personal beliefs and knowledge about the necessity of their prescribed medication but also by patient characteristics and how patients make an effort to remember to take their medications (Ownby et al., 2006). Nevertheless, elevated medication burden also increases risk for medication interactions (Palleria et al., 2013) and potentially inappropriate medication use (Nothelle et al., 2017). Therefore, efforts to alleviate medication burden namely by the selection of medications that treat more than one underlying condition, combined indications with single medication or fixed-dose combination medications (FDC), when possible, should be considered.

The overall MRCI score in this study was comparable to that of a previous study specific to patients with T2DM with similar criteria where only those chronic medications filled for at least 90 days were considered in the analysis (Boye et al., 2020). The diabetes-specific MRCI accounted for 52.3% of the overall MRCI score in this present study. Similar results was found in prior studies (Libby et al., 2013; Rettig et al., 2013) where the MRCI score was found to be greater for the group with diabetes than for the group with hypertension despite no difference in the mean number of medications. This indicates that to a certain extent, the complexity components for antidiabetic medications contribute much more to regimen complexity than sheer medication count. Antidiabetic regimens can be a mixture of oral and injection; inclusion of insulin in the treatment regimen adds to the score of regimen complexity. The application of MRCI tool to quantify pharmacotherapy complexity has been previously demonstrated; however, there are still inconclusive evidence on the appropriate cut-points of MRCI score to distinguish level of regimen complexity (Ferreira et al., 2015; Wimmer et al., 2016; Morillo-Verdugo et al., 2019). As such, meaningful comparisons across studies are difficult until the range of plausible MRCI is established.

The findings that the MRCI score was inversely associated with good glycemic control in the current study are consistent with those of previous reports specific to patients with T2DM (Pollack et al., 2010; Yeh et al., 2017; Ayele et al., 2019). However, it is important to take note that approximately 45% of the patient-level score was derived from non-antidiabetic medications such as antihypertensive medications and lipid-lowering agents. Despite the increase of the patient-level MRCI score by these medications, some of these medications may be associated with secondary effects on the glycemic levels. For instance, non-antidiabetic medications such as beta-blockers, salicylates and angiotensin-converting enzyme inhibitors have been reported to contribute to medication-induced hypoglycemia, even in individuals without diabetes (Vue and Setter, 2011). As expected, we found a positive association between good medication adherence and improvement in glycemic control. This is in keeping with those reported in several studies that greater adherence was associated with improved glycemic control (Schectman et al., 2002; Ho et al., 2006; Lin et al., 2017). One of the viable strategies that can be employed to reduce the regimen complexity is by reducing the frequency of the prescribed medications. It has been reported that simplification of medication dosing has a significant positive effect on adherence (Coleman et al., 2012; Weeda et al., 2016). However, increased medication burden may be unavoidable in most chronic conditions (Ong et al., 2020). Therefore, it is vital to have a regular review of patients’ medication regimens to ensure that unnecessary and redundant medications are discontinued.

The findings of this study have to be considered within several limitations. First, this study focused on T2DM patients treated at public primary clinics and hence may not present the entire population of individuals with T2DM. However, a large proportion of diabetes patients seek treatment at public health facilities (Hussein et al., 2015; Sivasampu et al., 2016) and the population included in the current study may reflect most of the target population, albeit not the whole. Second, the analysis did not consider over-the-counter medications, vitamins or supplements when examining patient medication regimens. These medications may or may not have the potential to contribute significantly to the overall complexity. Thus, the overall MRCI might be underestimated. Third, medication use was proxied by medication dispensing data. As such, we were unable to confirm whether patients were taking the medications when a prescription was filled. Further, refilling a prescription on time does not necessarily mean that a patient is taking the drug correctly. This may overestimate adherence to treatment for some patients. Another limitation was that only one HbA1c measurement was used for the assessment of the relationship between medication regimen complexity measure and medication adherence. Nevertheless, we are confident with the findings because most of the study findings are consistent with many other related studies.

## Conclusion

High medication count and regimen complexity were associated with lower odds of attaining good glycemic control while inverse association was observed between medication adherence and HbA1c level. These findings also suggest that medication adherence is likely the mediator in the association between regimen complexity and glycemic control. Our study showed that MRCI can be utilized as an objective proxy in addressing the complexity inherent within a medication prescription. Thus, it can be used to help with clinical decision support by identifying patients with complex pharmacotherapy regimens who can then be evaluated further before remedial action can be taken. This includes medication regimen simplification, reducing pill burden, medical reminder devices, closer clinical monitoring and clinical interventions if required as well as boosting patients’ understanding of prescribed medications to improve adherence and achieve optimum outcomes in patients with T2DM and ease of self-care.

## Data Availability

The original contributions presented in the study are included in the article/Supplementary Materials, further inquiries can be directed to the corresponding author.
