# Supplementary material for: Medication Regimen Complexity and Medication Burden Among Patients With Type 2 Diabetes Mellitus: A Retrospective Analysis
Source: Front Pharmacol. 2022 Mar 21;13:808190. doi: 10.3389/fphar.2022.808190 (PMC8978326; doi:10.3389/fphar.2022.808190)
Supplement: Supplementary file 1 [file DataSheet1.docx]

**Appendix**

Table S1 Association between medication parameters and HbA1c level using multiple linear regression

|  | **Patient-Level** | | | | **Diabetes-Specific** | | | |
| --- | --- | --- | --- | --- | --- | --- | --- | --- |
|  | **Standardised regression coefficient**  **(95% CI)** | **t** | **P value** | **Adjusted R^2^ of the model** | **Standardised regression coefficient**  **(95% CI)** | **t** | **P value** | **Adjusted R^2^ of the model** |
| **Medication count** | 0.152 (0.08, 0.22) | 4.46 | <0.001 | 0.118 | 0.404 (0.28, 0.53) | 6.79 | <0.001 | 0.125 |
| **MRCI score** | 0.110 (0.08, 0.14) | 8.23 | <0.001 | 0.157 | 0.284 (0.25, 0.32) | 17.8 | <0.001 | 0.239 |
| **Adherence (PDC)** | -0.677 (-1.34, -0.01) | -2.15 | 0.047 | 0.1094 | -0.150 (-0.86, 0.56) | -0.45 | <0.001 | 0.104 |

Note: Model was run separately for (i) medication count, (ii) MRCI score, and (iii) adherence. Adjusted for age (years), gender (male/female), ethnicity (Malay/Chinese/Indian/others), hypertension (no/yes), hyperlipidaemia (no/yes), body mass index (kg/m2), location of primary care clinic (rural/urban), duration of diabetes (years). p-value significant at <0.05.

Abbreviations: CI, confidence interval; MRCI, medication regimen complexity index; PDC, proportion days covered.

Table S2 Association between medication parameters and HbA1c level (≤7.5%) of study cohort from “EnPHC-Eva: Facility” Malaysia, 2018-2019

|  | **Unadjusted** | **Adjusted** | | |
| --- | --- | --- | --- | --- |
|  | **OR (95% CI)** | **OR (95% CI)** | **p-value** | **R^2^** |
| **Patient-level** |  |  |  |  |
| Medication count | 0.91 (0.87, 0.91) | 0.87 (0.82, 0.92) | <0.001 | 0.0887 |
| MRCI score | 0.91 (0.89, 0.92) | 0.90 (0.87, 0.92) | <0.001 | 0.1156 |
| Adherence (PDC) | 1.48 (0.88, 2.46) | 1.94 (0.96, 3. 90) | 0.065 | 0.0832 |
|  |  |  |  |  |
| **Diabetes-specific** |  |  |  |  |
| Medication count | 0.56 (0.51, 0.63) | 0.62 (0.55, 0.70) | <0.001 | 0.0986 |
| MRCI score | 0.70 (0.68, 0.72) | 0.72 (0.69, 0.76) | <0.001 | 0.1850 |
| Adherence (PDC) | 0.82 (0.49, 1.36) | 1.19 (0.54, 2.60) | 0.664 | 0.0818 |

Note: Model was run separately for (i) medication count, (ii) MRCI score, and (iii) adherence. Estimation using univariable (unadjusted) and multivariable logistic regression adjusted for age (years), gender (male/female), ethnicity (Malay/Chinese/Indian/others), hypertension (no/yes), hyperlipidaemia (no/yes), body mass index (kg/m2), location of primary care clinic (rural/urban), duration of diabetes (years). p-value significant at <0.05. The variance inflation factors (VIFs) for all variables were less than 2 in all models.

Abbreviations: OR, odds ratio; CI, confidence interval; MRCI, medication regimen complexity index; PDC, proportion days covered.
